# Exploring the native pulp and paper sludge microbiome to inspire new biotechnologies for waste minimization

**DOI:** 10.1128/spectrum.01359-26

**Published:** 2026-06-09

**Authors:** Pakinee Thianheng, Kurt Louis Schroeter, Johan Larsbrink, Lauren Sara McKee

**Affiliations:** 1 Division of Glycoscience, Department of Chemistry, KTH Royal Institute of Technology 7655, Stockholm, Sweden; 2 Wallenberg Wood Science Center 521086, Stockholm, Sweden; 3 Department of Life Sciences, Chalmers University of Technology 11248, Gothenburg, Sweden; 4 Wallenberg Wood Science Center, Gothenburg, Sweden; Connecticut Agricultural Experiment Station, New Haven, Connecticut, USA

**Keywords:** marker gene profiling, sludge, pulp and paper, bioremediation, microbiome

## Abstract

Thousands of metric tonnes of diverse sludge wastes are generated annually in the pulp and paper industry. Due to a high moisture content and an abundance of inorganic material, many types of sludge are hard to recycle and instead accumulate in landfills, causing environmental damage. During storage, forestry sludge waste appears recalcitrant to natural attenuation, indicating limited degradation of wood- and process-derived fibers and polymers by environmental microbes. Intentional enzymatic or microbial hydrolysis of carbohydrates within the sludge may, however, be a feasible approach to reduce waste volume and prevent transfer to landfill. Here, we show that a previously validated biomass-degrading enzyme cocktail lacks efficacy on a metal-rich sludge obtained from a Swedish pulp and paper mill, possibly due to enzyme inhibition. Hypothesising that microbes dwelling within sludge may host enzymes better adapted to this complex contaminated substrate, we assessed whether a native sludge microbiome could be identified, and whether it degrades carbohydrates during incubation in microcosms. Marker gene profiling revealed diverse bacterial and fungal communities undergoing genus-level changes over time, and the most abundant species could be enriched via serial cultivation with pulp-derived carbon sources. Complementary chemical analyses showed that biopolymers were largely removed after a 10-week incubation, leading to sludge solubilization and volume reduction. This confirms the capacity for fiber degradation by native microbiomes and suggests the waste as a potential source of microbes and enzymes capable of sludge polymer degradation, the mechanism of which remains to be explored, but which could reduce the need for future landfilling.

**IMPORTANCE:**

According to European and Swedish guidelines, the top priority in waste handling is prevention, followed by reuse, recycling, energy recovery, and, as a last resort, landfill. While effective in municipal contexts, these guidelines are difficult to apply to pulp and paper industries when managing heterogeneous sludge wastes. Process-derived sludges are hugely abundant but have low economic value as their high moisture content prevents combustion, and the complex mixture of organic fibers prevents metal recovery. According to industrial reports, less than 10% of sludge is used for energy, and under 50% is recycled. Our results demonstrate that biological treatment of sludge could be a method of waste reduction to reduce landfilling, specifically targeting hygroscopic carbohydrate-based polymers. Mapping the microorganisms in this under-explored industrial waste material, using combined “omic” technologies and chemical analysis, lays a foundation for discovering robust organisms and enzymes that withstand harsh conditions, such as low water activity and high metal content.

## INTRODUCTION

The pulp and paper industry is of global importance, converting diverse forestry feedstocks into products, such as pulp, paper, and packaging materials. The global wood pulp market is estimated to consume at least 200 million metric tonnes of wood each year, and the need for paper products and cardboard packaging materials continues to increase. Indeed, wood pulping for paper and board is one of the largest industrial sectors in Sweden, a dominant player in the global market. According to the Swedish Forest Industries Federation, the country has 40 pulp and paper mills, 80 sawmills, and 105 companies closely connected to the production of pulp, paper, or timber goods (1). Although the industry is based on renewable resources, it leads to an annual generation of many thousands of tonnes of poorly recyclable waste, such as tree bark and diverse process-derived sludges, which represent the last tiny fraction of harvested forest biomass that is still not industrially circularized in Sweden (2).

One type of sludge produced during paperboard manufacturing is precipitation sludge (PS), generated from the metal-induced precipitation of low-quality fibers that would otherwise compromise the quality of the final product. In a single mill, PS can be generated at around 8,000 tonnes per year, according to industry operatives (personal correspondence). PS has a high moisture content, making it unsuitable for energy recovery through combustion (3) due to the excessively high costs of dewatering and the need for specialized combustion facilities. Additionally, the presence of a mixture of metals and other elements along with wood fibers and biopolymers restricts recyclability, leading to the common disposal of PS in landfills or waste piles on private land. A major associated concern is the leaching of metals and potentially toxic elements into the surrounding nature, which inevitably causes environmental damage (4). In addition, there is concern within the industry that current Swedish legal restrictions against municipal landfilling may be extended to the private sector, creating a sense of urgency and a desire to minimize the volume of PS sent to landfill. According to our correspondence with industry stakeholders, even a 5%–10% reduction in sludge volume could significantly improve the economy and practicality of waste management.

Although PS is rich in inorganic matter and metals, it also contains abundant organic components, such as starch, cellulose, hemicelluloses, and lignin, deriving both from the wood itself and from additives in the pulping process (5, 6). While *in situ* treatment of existing landfill and waste piles is an ongoing industrial challenge, we propose that the aforementioned biopolymers may be amenable to hydrolytic degradation by enzymes and/or living microbes, thus solubilizing the waste, minimizing its bulk volume, and preventing transfer to landfill in the first place.

Many hydrolytic enzyme cocktails have been developed for the breakdown of complex biomass (7–9), and in this work, we assess one such cocktail, finding it to be of limited efficacy compared to its previous validations on non-contaminated substrates (10, 11). Hypothesizing that metals or other inhibitory compounds within PS may be responsible for the lack of enzyme activity, we speculated that there may be a resident PS microbiome that carries enzymes better adapted to biopolymer breakdown in these specific substrate conditions (i.e., metal contaminants, high solid content, low water activity). Using experimental microcosms, we explored the degradation of cellulose, starch, and xylan over several weeks in an anoxic environment to simply determine whether microbial growth and carbohydrate degradation could take place in PS. In general, the efficacy of microbial degradation of biomass depends on the composition, dynamics, and functional capacity of the microbial community (12, 13). The presence of several types of carbon sources in environments like sludge waste creates a complexity generally expected to enhance microbial diversity and species richness (14–18), although when components in the substrate are toxic, microbial diversity may be reduced until these have been consumed or detoxified (19). The resulting uncertainty makes it hard to predict the sludge-degrading capacity of any microbiome extant in PS (20, 21), so more information is needed before enrichment cultures can be developed for *in situ* waste minimization.

Here, these aspects of microbial community dynamics were explored in PS-based microcosms, some of which were supplemented with small amounts of sludge-mimicking carbon sources, in a first principles assessment of whether there is a native PS microbiome that may be a source for microbes capable of PS biopolymer degradation. Next-generation long-read marker gene DNA sequencing was employed to analyze changes in the fungal and bacterial communities, alongside a chemical examination of shifts in the organic composition of the sludge. We aimed to enhance understanding of microbial interactions and adaptations in an industrial environment, hoping to contribute to the eventual goal of improved waste minimization strategies for more efficient breakdown of organic components.

## MATERIALS AND METHODS

### Sludge samples

Precipitation sludge (PS, 66% moisture content, pH 6.96) generated during the production of paperboard was collected from a pulp and paper mill in the middle of Sweden. The sludge was stored in Ziploc bags and transported to the Stockholm CAZyme Lab at KTH Royal Institute of Technology. Prior to the preparation of experimental microcosms, the sludge was exposed to outdoor conditions for a period of 2 weeks. This procedure aimed to simulate the normal process of inoculation by environmental microbes, which typically occurs when sludge is stored outside or disposed of in landfills. The moisture content in PS was measured by drying five PS samples at 60°C for a week and was calculated using equation 1, where W1 is the weight of the empty container, W2 is the weight of the container plus the wet sample, and W3 is the weight of the container plus the dry sample.


(1) $$Moisture\;content\;\left(\%\right)=\frac{W2-W3}{W2-W1}\times 100.$$

To determine the metal content, a 50 g sample of PS was sent to Eurofins Water Testing Sweden AB for elemental composition analysis. Aluminum (Al), iron (Fe), silica (Si), and calcium (Ca) in the sludge were measured after sample digestion using a mixture of hydrofluoric, nitric, and hydrochloric acids, following the Swedish standard SS-EN 13656:2020.

### Carbon sources used

The following four carbon sources were used in this study: starch from corn (Sigma-Aldrich, Germany), Avicel-PH101 cellulose (Fluka Chemie, Germany), xylan from beech wood (Megazyme, Ireland), and alkaline lignin (Sigma-Aldrich, Germany).

### Enzymatic hydrolysis test

An enzyme cocktail from *Trichoderma reesei* was used to test the biodegradation of PS (11). Hydrolysis reactions (1 mL total volume) were set up in 2-mL screw-top tubes with 10% dry content in 100 mM citrate buffer, pH 5.0, and incubated at 50°C for up to 72 h in a hybridization incubator with rotating agitation. The standard 1× enzyme loading represents 10 mg enzyme per 1 g dry biomass: enzyme loadings of 1× and 5× were tested. In addition, experiments were performed both with and without the inclusion of 0.02% sodium azide to inhibit microbial metabolism of released sugars, which confounded measurements in early tests. Following incubation, the reactions were centrifuged for 5 min at 12,000 × *g,* and the supernatant and solids were boiled separately for 10 min to inactivate the enzymes. The DNSA (3,5-dinitrosalicylic acid) assay was used to quantitatively measure the amount of reducing sugar (22), measured as glucose equivalents using a standard curve of glucose. Control reactions with the enzyme cocktail substituted with an equivalent volume of dH_2_O were used as blanks. An equivalent volume of enzyme cocktail was instead added to the DNSA reagent prior to adding the supernatant to account for potential reducing power from the cocktail itself. Triplicate reactions of all samples were performed.

### Microcosm preparation and incubation

PS microcosms were prepared under five different conditions, all containing 30 g of PS. The five conditions included (i) no supplementation, (ii) addition of 0.15 g starch, (iii) addition of 0.15 g cellulose, (iv) addition of 0.15 g xylan, and (v) addition of 0.15 g lignin, i.e., a final concentration of 0.5% (wt/wt) supplemented carbon source. Each condition was prepared in duplicate. To these mixtures were added 10 mL of M9 medium (23), followed by static incubation at 25°C for 10 weeks. During the 10-week incubation, 5 g samples were collected from each condition every 2 weeks in duplicate, and pH was monitored at the time of sample collection. Due to the opening of the microcosm jars, air was introduced during sampling, preventing fully anaerobic conditions from establishing. Likewise, the landfill piles at pulp mill sites are often regularly turned over to prevent full anaerobic conditions from developing (personal correspondence). At each sampling point, one set of duplicates was stored at −80°C for subsequent microbial community analysis, while the other was sterilized by exposure to UV after spreading to maximize the exposed surface area, and subsequently dried at 37°C for composition analysis.

### Enrichment cultivation

After 2 weeks of incubation, 1 g from each sludge microcosm was transferred into 50 mL of M9 medium, supplemented with 0.5% (wt/vol) of the respective supplemented carbon source. Cultivation in these semi-liquid conditions continued at 25°C for 2 weeks under anoxic conditions without shaking. After the initial 2-week period, a 1 mL sample of the culture was transferred to 50 mL of fresh M9 medium containing 0.5% of the same carbon source. This step was repeated four times. During each enrichment, a sample of the cell pellet was collected by centrifugation at 4°C, 6,200 × *g* for 10 min, then stored at −80°C for subsequent microbial community analysis.

### Carbohydrate composition analysis

Carbohydrate content in the sludge was investigated for the original PS and for each microcosm sampled at biweekly intervals. The starch content was quantified using the Total Starch Kit (Megazyme), according to the manufacturer’s instructions. Hemicellulose and cellulose contents were determined by sequential hydrolysis of samples with trifluoroacetic acid (TFA), followed by sulfuric acid. The hydrolysis started from 5 mg of dry sludge mixed with 1 mL of 2 M TFA, heated to 120°C for 3 h. After cooling, the TFA was evaporated overnight, and 1 mL of Milli-Q water was added, followed by a further 1:10 dilution in water. The samples were filtered through 0.22 µm nylon filters into 2-mL vials for analysis by high-performance anion exchange chromatography with pulsed amperometric detection (HPAEC-PAD). This same method of TFA hydrolysis and HPAEC-PAD analysis was used to study the carbohydrate composition in residual solids left after PS incubation with an enzyme cocktail. For HPAEC-PAD analysis, a Dionex ICS-6000 high-performance liquid chromatography system, operated by Chromeleon software v7 (Dionex), equipped with a Dionex CarboPac PA1 column (Thermo Scientific), was used to identify and quantify monosaccharides as described previously (24).

The carbohydrate portion of the residual sludge material left undigested by TFA hydrolysis mainly comprised crystalline cellulose and was next subjected to sulfuric acid hydrolysis. A volume of 125 µL of 72% sulfuric acid was added to the residue and incubated at room temperature for 3 h with frequent stirring. Next, 1.375 µL of Milli-Q water was added, and the mixture was heated to 100°C for 3 h. After cooling, the solution was diluted 1:10 with water, filtered through 0.22 µm nylon filters, and transferred into 2-mL vials for HPAEC-PAD analysis. The amounts of glucose and xylose were quantified by comparison with monosaccharide standards (Fig. S2). Cellulose content is presented as non-starch glucose, and xylan content is presented as the concentration of xylose.

### Lignin content analysis

Lignin content was determined by gravimetric analysis following sulfuric acid hydrolysis. The process began by adding 0.5 mL of 72% sulfuric acid to 50 mg of dry sludge. The samples were incubated at room temperature under shaking conditions at 150 rpm for 3 h. After incubation, 5.5 mL of Milli-Q water was added and mixed thoroughly before heating in a thermoblock at 100°C for 3 h. Following heating, the hydrolyzed samples were cooled and collected on filter paper using a gravimetric technique: a vacuum pump (Millivac-Maxi Pump, Millipore) was used to draw the solution through the filter paper. The retained solids were then dried at 60°C. Finally, lignin content was calculated based on the weight of the dry particles.

### Microbial community analysis

Microbial marker gene analysis was conducted by DNASense (Denmark, https://dnasense.com/) to identify bacteria and fungi. Sludge microcosm samples (1–2 g) were stored at −80°C before shipment to DNASense under cold storage conditions. DNA was extracted using the FastDNA Spin Kit for Soil (MP Biomedicals, USA).

For bacterial community profiling, PCR amplicon libraries were prepared with primers [8F] AGRGTTYGATYMTGGCTCAG and [1391R] GACGGGCGGTGWGTRCA. Sequencing libraries were prepared with the SQK-LSK114 kit (Oxford Nanopore Technologies, UK) and loaded onto a PromethION R10.4.1 flowcell. Sequencing was performed using MinKNOW 24.02.10 software, and reads were base-called and demultiplexed with MinKNOW Dorado 7.3.9. Filtered reads (320–2,000 bp, phred score >17) were mapped to the SILVA 16S/18S rRNA 138 SSURef NR99 database using minimap2 v2.24-r1122. Only reads with query sequence length deviations <5% from alignment length were retained. Low-abundant operational taxonomic units (OTUs) (<0.01% of total mapped reads) were excluded for data denoising.

For fungal community profiling, amplicon libraries for the fungal ITS2 (fITS2-C) region were prepared using primers [ITS2-9] TACACACCGCCCGTCG and [ITS4] TCCTSCGCTTATTGATATGC. DNA sequencing and reads were processed as described above. Filtered reads (320–2,000 bp, phred score >17) were mapped to the QIIME-formatted UNITE database release 9.0 with minimap2 v2.24-r1122. Mapping results were filtered for alignment lengths >125 bp and mapping quality >0.75. Low-abundant OTUs making up <0.01% of total mapped reads were excluded for data denoising as well.

### Statistical analysis

Alpha diversity of bacteria and fungi communities was determined using the Shannon index, Simpson index, and Chao1, plotted by Python 3.13.1, with the modules as listed: seaborn 0.13.2, pandas 2.2.2, and numpy 1.26.4. Significant statistical differences in alpha-diversity between samples were analyzed by the Shapiro-Wilk test to check data normality, followed by the Kruskal-Wallis test and Pairwise Wilcoxon test for normally distributed data (Shapiro-Wilk, *P* value > 0.05) or ANOVA test, followed by Tukey’s HSD test for abnormally distributed data (Shapiro-Wilk, *P* value < 0.05). All statistical analyses were performed using RStudio-2024.12.0-467.

## RESULTS AND DISCUSSION

**Fig 1:**
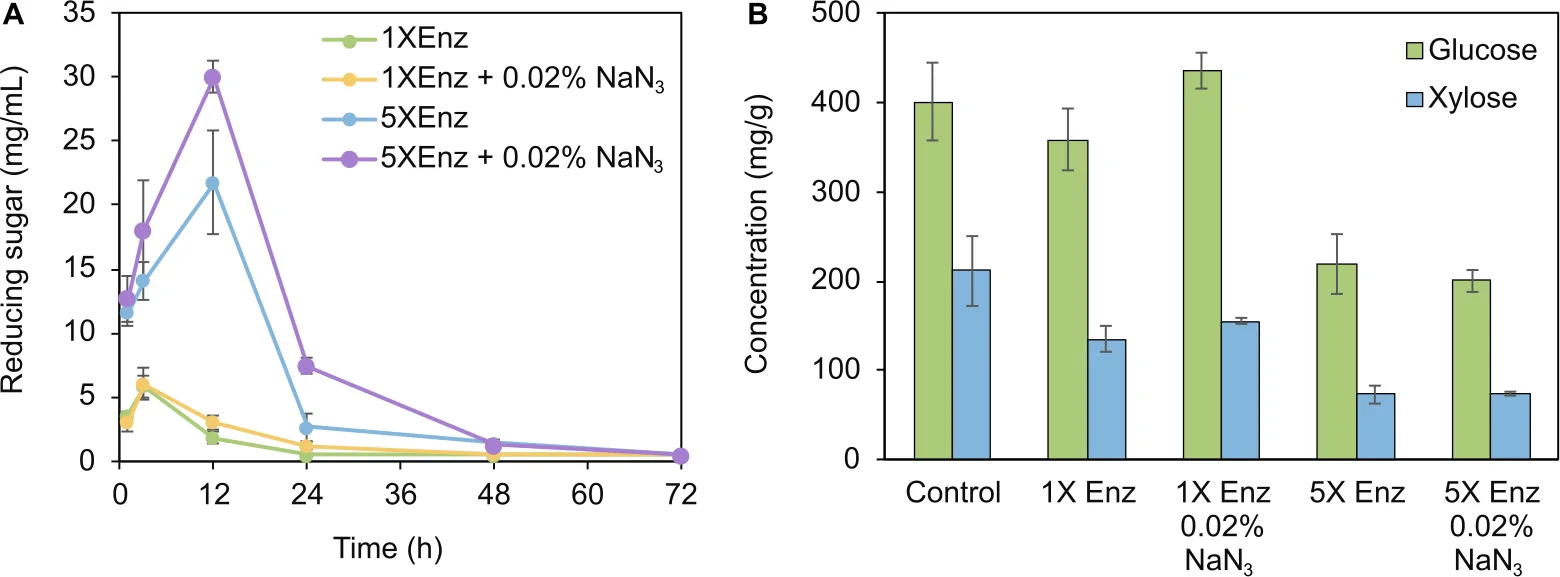
Sludge breakdown by the enzyme cocktail. (**A**) Total reducing sugar released from PS over a 72-h incubation period. One time of enzyme loading is 10 mg of enzyme cocktail per gram of PS. (**B**) Total amounts of glucose and xylose released by acid hydrolysis of the residual solids remaining after enzyme hydrolysis. Average values are shown, with error bars representing the standard error of the mean for panel **A** and the standard deviation for panel **B**.

PS is a light brown, heterogeneous, fibrous material (Fig. S1). A moisture content of 66% gives it a semi-solid consistency, and it appears quite dry to the eye, due to the absorbent nature of the fibers, organic polymers, such as wood cellulose and lignin, as well as cationic starch added during the pulping process (25). Our analyses show that the PS we obtained was rich in process-derived metals, with concentrations of 130 mg of Fe, 96 mg of Si, 54 mg of Al, and 16 mg of Ca per gram of sludge. Furthermore, PS consisted of approximately 5% starch, 24% cellulose, 7% xylan (the only detectable hemicellulose), and 26% lignin, based on dry weight (Fig. S2). As our goal is to eventually develop biological techniques to minimize PS volume, we first tested enzymatic hydrolysis of the carbohydrate fraction, hoping to break down most of the starch, cellulose, and xylan into monosaccharides that may be of use in a sugar platform (26, 27). For this, a recently developed enzyme cocktail, previously shown to be comparable in efficacy to commercial cocktails (11), was tested. The recommended enzyme loading (1×: 10 mg enzyme per 1 g of biomass) was first used, but minimal release of reducing sugars was observed (Fig. 1A). We proposed that this may be due to scavenging of released sugars by living microbes within the sludge, so enzyme experiments were repeated with the inclusion of 0.02% sodium azide to inhibit microbial growth. Still, at 1× enzyme loading, no significant release of reducing sugars was observed, and analysis of residual solids revealed significant amounts of glucose- and xylose-containing polysaccharides in the remaining unhydrolyzed solid material (Fig. 1B). Thus, enzyme loading concentration was increased to 5×, leading to an improvement in reducing sugar yield. Residual solids analysis showed that the cellulose:xylan ratio appeared roughly the same in the more hydrolyzed sample (5× enzyme loading) compared to the starting material, indicating that the enzyme cocktail removed both polysaccharides at a similar rate (Fig. 1B). Nonetheless, much of the original polysaccharide was still present after enzyme incubation, indicating a poor degradative performance by the biocatalytic cocktail even at the higher enzyme loading, which represents a process with poor economic feasibility.

We speculated that the lack of hydrolytic efficacy from this validated enzyme cocktail might be due to the complexity of the PS substrate, or that these enzymes may be inhibited by the metals or other compounds present. This, and the observation that more reducing sugars could be detected when sodium azide was included in enzyme assays, motivated us to explore whether a native microbiome residing in PS, adapted to conditions in the sludge, might harbor enzymes more capable of breaking down the polysaccharides. Thus, we initiated a first-principles study to explore the microbes that colonize PS and their ability to metabolize carbohydrates therein.

We created a series of microcosms to encourage microbial growth in relatively stable conditions. The original pH of the PS was measured at 6.96. At the onset of microcosm incubation, the initial pH in all microcosms was ~7.2 due to the addition of M9 medium. However, the pH decreased to 6.2–6.7 following 2 weeks of incubation. Subsequently, it showed a slight increase, returning to 7.0–7.2 after 10 weeks (Fig. S3). This likely indicated the presence of acidogenic microorganisms growing and releasing acidic by-products during the first 2 weeks.

### Reduced abundance of wood pulp biopolymers during microcosm incubation

**Fig 2:**
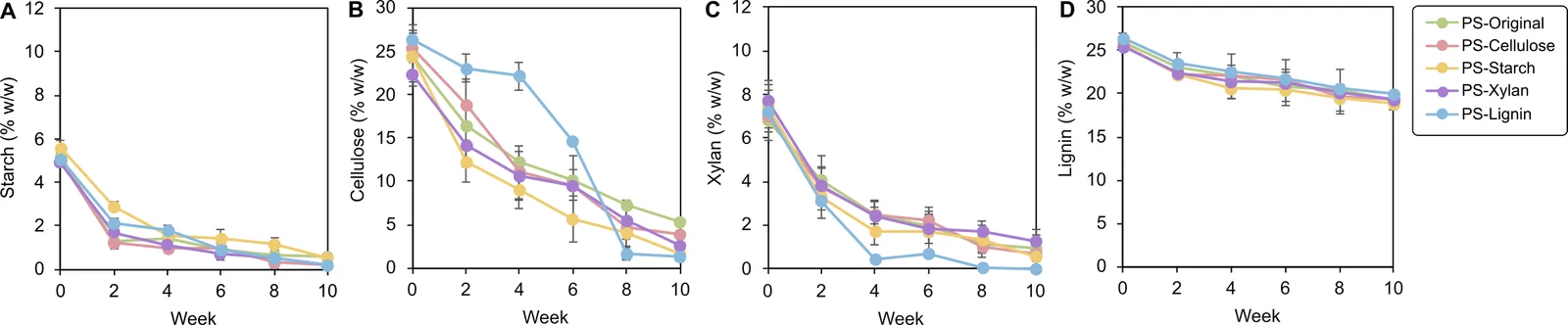
The changes in organic content in PS microcosms incubated for 10 weeks, for (**A**) starch, (**B**) xylan, (**C**) cellulose, and (**D**) lignin. The amounts are shown as percentages (wt/wt) of total sludge based on dry weight, with the different conditions indicated as PS-Original for unsupplemented PS, and PS-Cellulose/Starch/Xylan/Lignin for the supplemented samples (0.5% wt/wt). Starch was measured by a starch kit. Cellulose and xylan were analyzed using acid hydrolysis-based methods and monitored via HPAEC-PAD. Lignin was analyzed using acid hydrolysis-based methods and measured by gravimetric analysis. Data are shown as averages from samples that were analyzed in triplicate, and error bars indicate standard deviation.

After 10 weeks of incubation, noticeable changes were observed in the sludge in all conditions. The material was visibly more homogeneous and dissolved, indicating the breakdown of large absorptive fibers (Fig. S1). For a quantitative analysis, concentrations of starch, cellulose, xylan, and lignin in the PS microcosms were monitored over 10 weeks (Fig. 2).

Across all conditions, starch exhibited the most rapid degradation, with nearly complete depletion within the first 2 weeks (Fig. 2A), indicating it is an easily accessible energy source for microbial growth. The supplementation of additional starch as a carbon source in growth medium at 0.5% (wt/wt) did not alter the trend of starch degradation, indicating that relevant microbial or enzymatic activities are not enhanced in those conditions. The degradation of xylan followed a similar trend to starch but at a slower pace; xylan content decreased substantially during the first 4 weeks and reached minimal levels by the sixth week across all conditions (Fig. 2B). Again, supplementation with the xylan carbon source did not alter the microbiome’s capacity for xylan removal, although the lignin-supplemented microcosms did remove xylan more rapidly during the first 4 weeks, leaving only ~2% xylan by the end of this early period (Fig. 2B). In contrast, the lignin-supplemented microcosm seemed to inhibit cellulose consumption during the first 4 weeks, compared to the other conditions. In the unsupplemented and non-lignin-supplemented samples, there was a steady reduction of cellulose throughout the 10-week incubation, whereas in the PS-Lignin sample, cellulose degradation was rapid only between 4 and 8 weeks, reaching a similar final level of cellulose removal at the end of the experiment (Fig. 2C). These findings suggest that the sludge microbiome in PS-Lignin, the microcosm that showed the clearest visual evidence of polymer solubilization (Fig. S1), initially prioritized the breakdown of xylan over cellulose, a distinct change in preference compared to other cultivation conditions. This insight may be helpful in designing future enzyme cocktails or microbial inocula for PS treatment. In all microcosms, there was only a minimal removal of lignin (Fig. 2D), likely due to the structural complexity and recalcitrance of this biopolymer. To promote lignin degradation, prolonged incubation or co-cultivation with exogenous lignin-degrading fungi (28, 29) is likely required.

### Snapshots of the overall microbial diversity in sludge microcosms

**Fig 3:**
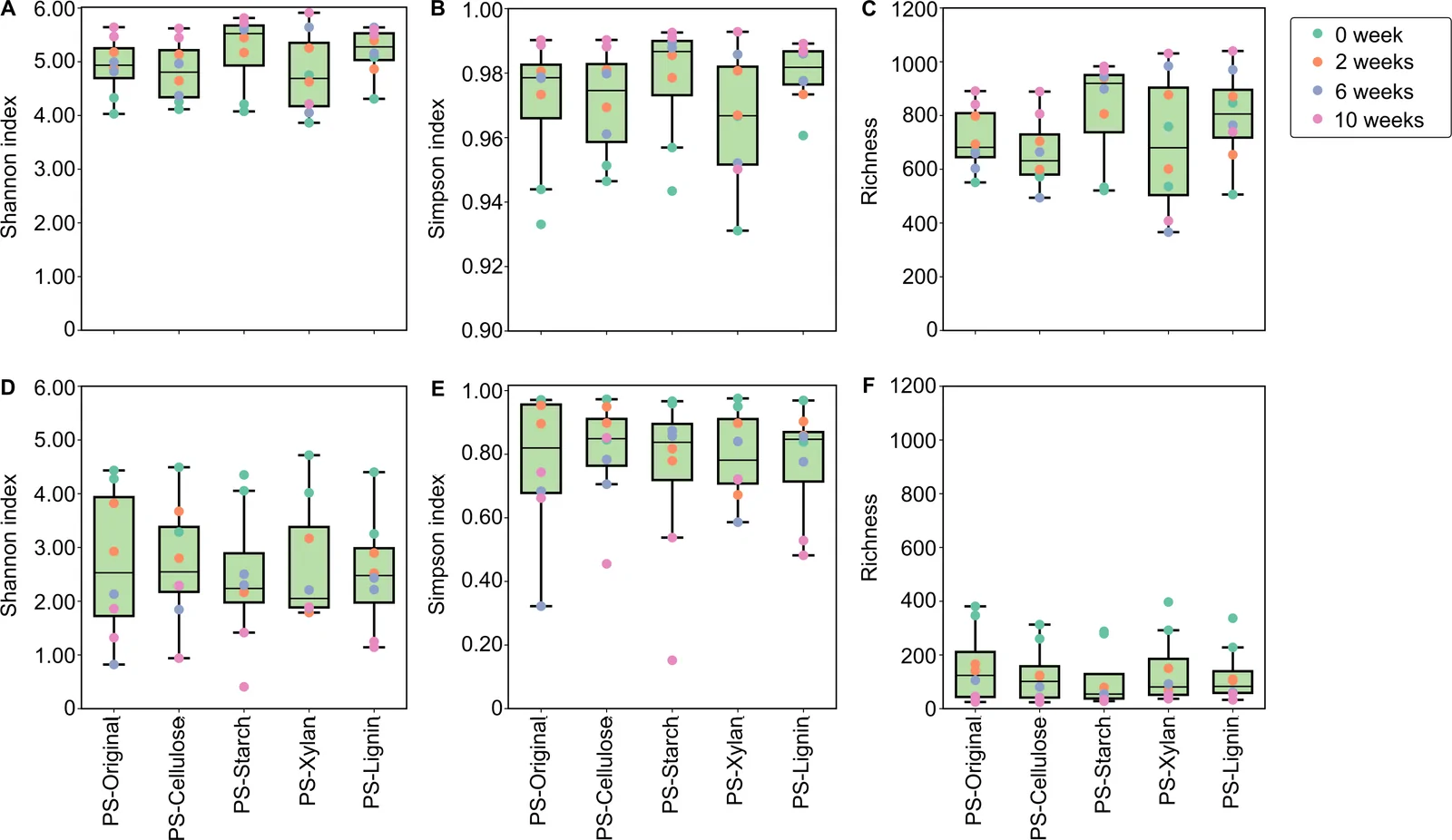
Box-and-whisker plots of alpha diversity over time for bacterial communities (Shannon index, **A**; Simpson index, **B**; Richness, **C**) and fungal communities (Shannon index, **D**; Simpson index, **E**; Richness, **F**) in PS incubated for 10 weeks. The statistical *P* values of pairwise comparisons are shown in supplement data (Tables S1 to S10). The sample names (e.g., PS-Cellulose) indicate the carbon source, if any, that was supplemented to the microcosm in growth medium at 0.5% (wt/vol).

The association of changes in PS with microbial growth was analyzed using next-generation long-read DNA sequencing. Trends in microbial diversity were revealed by measuring the Shannon index, Simpson index, and species richness. At the beginning of incubation, the Shannon indices of bacterial diversity in PS were 3.86–5.09 across all conditions (Fig. 3A), showing that no significant differences were induced immediately following the supplementation of carbon source (Kruskal-Wallis *P* value = 0.448; see Table S1 for *P* values of all pairwise comparisons). After 10 weeks of incubation, the Shannon indices increased to 4.21–5.91 across all carbon sources (Fig. 3A), still with no significant differences between conditions (Kruskal-Wallis *P* value = 0.359, Table S2). The Shannon indices of bacterial diversity in PS increased significantly over time for every condition (Kruskal-Wallis *P* value = 0.0002, Table S3), and Simpson indices displayed the same trend (Fig. 3B, Kruskal-Wallis *P* value = 2.964e-05, Table S4). Moreover, the species richness of bacteria in PS became significantly higher over time comparing week 0 and week 10 (ANOVA *P* value = 0.0413 and Tukey HSD *P* value = 0.0248), with no significant differences between carbon sources (Fig. 3C, ANOVA *P* value = 0.3487, Table S5). These analyses indicated that the bacterial communities in PS became more diverse over time in all conditions.

The Shannon index of fungal diversity in PS presented similar trends to that of bacterial diversity, being 3.23–4.72 at the beginning of incubation, with no significant differences between carbon sources (Fig. 3D, ANOVA *P* value > 0.05, Table S6). After 10 weeks of incubation, the Shannon index of fungal diversity declined to 0.41–2.29 with no significant differences between carbon sources (ANOVA *P* value > 0.05, Table S7). The Simpson index and richness showed the same trend, with no significant differences across carbon sources compared at the same time points (Fig. 3E and F, Kruskal-Wallis *P* value = 0.9979). Consequently, Shannon and Simpson indices and species richness of fungal diversity decreased over time, comparing week 0 and week 10 (Tukey HSD *P* value = 0.0000; Wilcoxon test *P* value = 0.0003, Tukey’s HSD, *P* value = 0.0000, Tables S8 to S10). In short, fungal communities in the original PS declined in richness during incubation in our experimental conditions, which apparently favored bacterial proliferation instead. Indeed, visual observation of microcosms did not reveal any obvious fungal colonization of the sludge surface. In different conditions, such as when sludge wastes accumulate outdoors, fungi may preferentially colonize the air-exposed surface of the piles while bacteria thrive in (semi-)anoxic conditions, such as those represented in our microcosms.

### The fungal community simplifies during microcosm incubation

**Fig 4:**
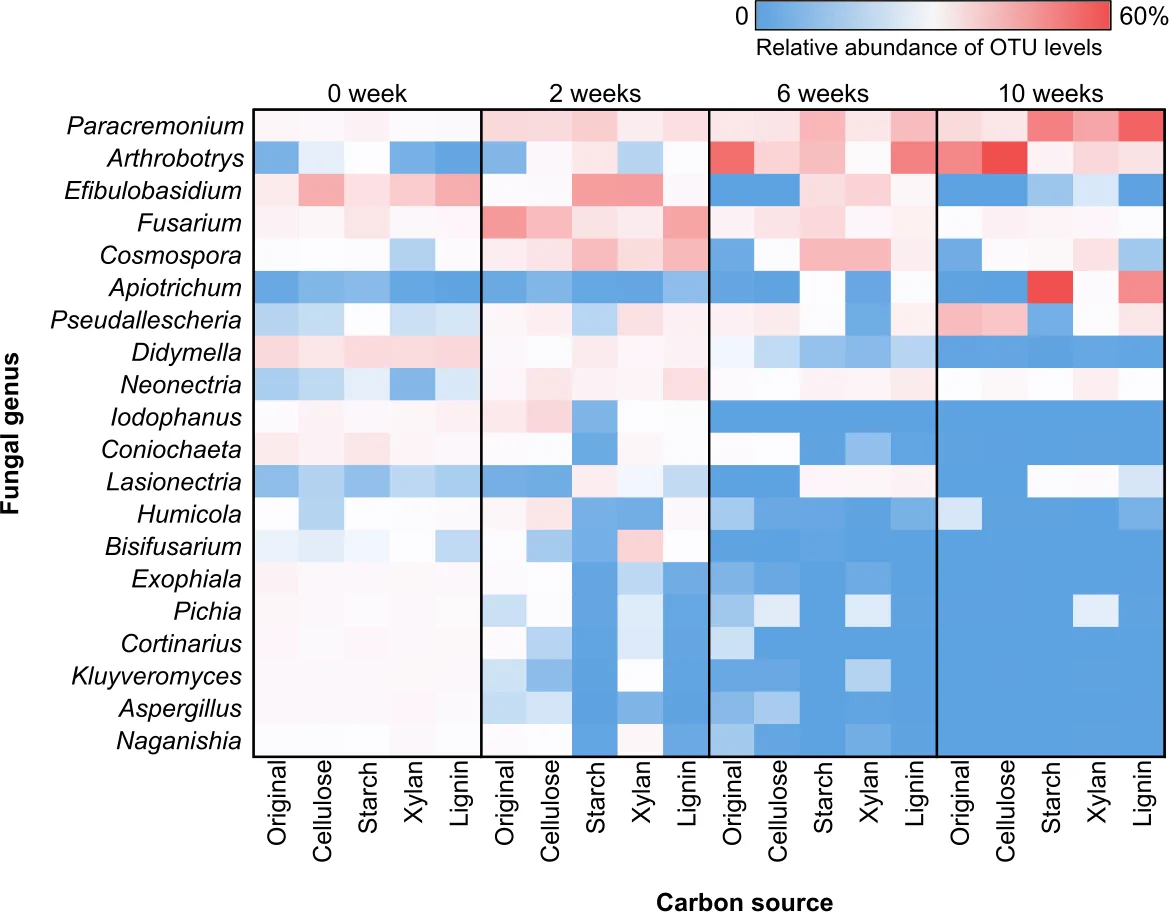
Heatmap illustrating the relative abundance of OTUs of 20 selected dominant genera of fungi in PS supplemented with different carbon sources (Original, Cellulose, Starch, Xylan, and Lignin) and at different incubation time points (0, 2, 6, and 10 weeks).

Long-read sequencing data enabled detailed analyses of fungal OTUs at the genus level, using the ITS gene for identification. At the onset of incubation (0 weeks), several fungal genera were found across all conditions (Fig. 4). After 2 weeks of incubation, changes in the fungal community began to emerge. Notably, a significant decrease in the relative abundance of OTUs of the genera *Exophiala*, *Pichia*, *Cortinarius*, *Kluyveromyces*, *Aspergillus*, and *Naganishia* was observed in xylan- and lignin-supplemented PS microcosms (Fig. 4). By 6 weeks, many other genera exhibited lower relative OTU abundances, while *Paracremonium*, *Arthrobotrys*, and *Fusarium* maintained notable levels (Fig. 4). By the end of the incubation period (10 weeks), a few specific fungal genera had become dominant. In particular, *Arthrobotrys* dominated in PS-Cellulose. *Arthrobotrys* sp. CX1 has previously been identified as a cellulose-gelatinizing fungus (30), suggesting that the unidentified *Arthrobotrys* species in PS might also play a role in cellulose degradation or modification. Its ability to solubilize cellulose may be useful in reducing the volume of sludge waste, a key wish for the industry (personal communication). Furthermore, the relative abundance of OTUs of the genus *Paracremonium* increased over time across all carbon sources and became the most prevalent in PS-Xylan and PS-Lignin (Fig. 4). It has been reported that *Paracremonium* sp. LCB1 was successfully used in co-cultivation with *Clonostachys compactiuscula* LCN1 to degrade lignin (31). The genus *Apiotrichum* was also dominant after 10 weeks in PS-Starch and PS-Lignin. A previous study showed an abundance of carbohydrate-active enzymes (CAZymes) in *Apiotrichum* species, with an average of 421 genes per genome (32), indicating a strong capacity for carbohydrate degradation, and suggesting the *Apiotrichum* genus as a candidate for sludge fiber degradation. These genera may be appropriate targets for future experiments aiming to isolate fungi for specific remedial applications.

### Bacterial community dynamics reflect changes in the sludge substrate

Bacterial communities in the incubated PS were studied by marker gene sequencing focused on the 16S rRNA variable regions V1–V8. At the start of the incubation period (0 weeks), when the concentrations of all polysaccharides were at their highest (Fig. 2A through C), bacterial communities across all PS samples exhibited similar structures, again indicating that there was not an immediate change due to the introduction of small amounts of supplemented carbon sources. The genera *Paludibacter, Propionicimonas, Cloacibacterium,* and *Saccharimonadales* were dominant during the initial stage of incubation. These are sugar-fermenting and acid-producing microbes (33–37), and their metabolic activity likely explains the observed drop in pH after 2 weeks (Fig. S2). The genus *Paludibacter* is known for its ability to utilize various sugars and produce acetate and propionate (35, 36). To date, only two species, *Paludibacter jiangxiensis* and *Paludibacter propionicigenes*, have been reported within this genus (https://lpsn.dsmz.de/genus/paludibacter), neither of which was an exact match for the OTU identified in our PS samples (Table S15), suggesting a novel *Paludibacter* species in this microbiome. The genus *Demequina* was also found in the original PS. Although most commonly found in coastal ecosystems (38), a novel species, *Demequina capsici*, was recently discovered to be a plant growth-promoting bacterium, suggesting more ecosystem diversity for the genus than previously known. The unidentified *Demequina* OTUs in the PS samples may also represent a novel species associated with plants or wood-associated environments (Table S15).

After 2 weeks of incubation, when starch and xylan had been significantly depleted and cellulose has become the more dominant polysaccharide (Fig. 2A through C), the bacterial communities showed more even distributions, with no single genus being particularly dominant. After 6 weeks, it was noticeable that the *Christensenellaceae_R-7_group* dominated in all conditions, while a reduction in relative OTU abundance was observed for *Paludibacter*, *Propionicimonas*, *Cloacibacterium*, *Saccharimonadales*, and *Demequina*. Indeed, after 10 weeks, when only small amounts of residual xylan and cellulose remained in the samples (Fig. 2A through C), *Christensenellaceae_R-7_group* was the predominant genus; its members have been shown to play a role as obligately anaerobic fermenters to produce acetate and other organic acids from sugars (39). Previous studies have reported that *Christensenellaceae_R-7_group* is typically associated with the gut microbiota (40–42), although it has been identified in sludges from swine farms (43). It has not been previously reported to be found in non-manure sludge like pulp and paper waste streams, and the PS-associated stains from this genus may represent as-yet unknown species (Table S15).

**Fig 5:**
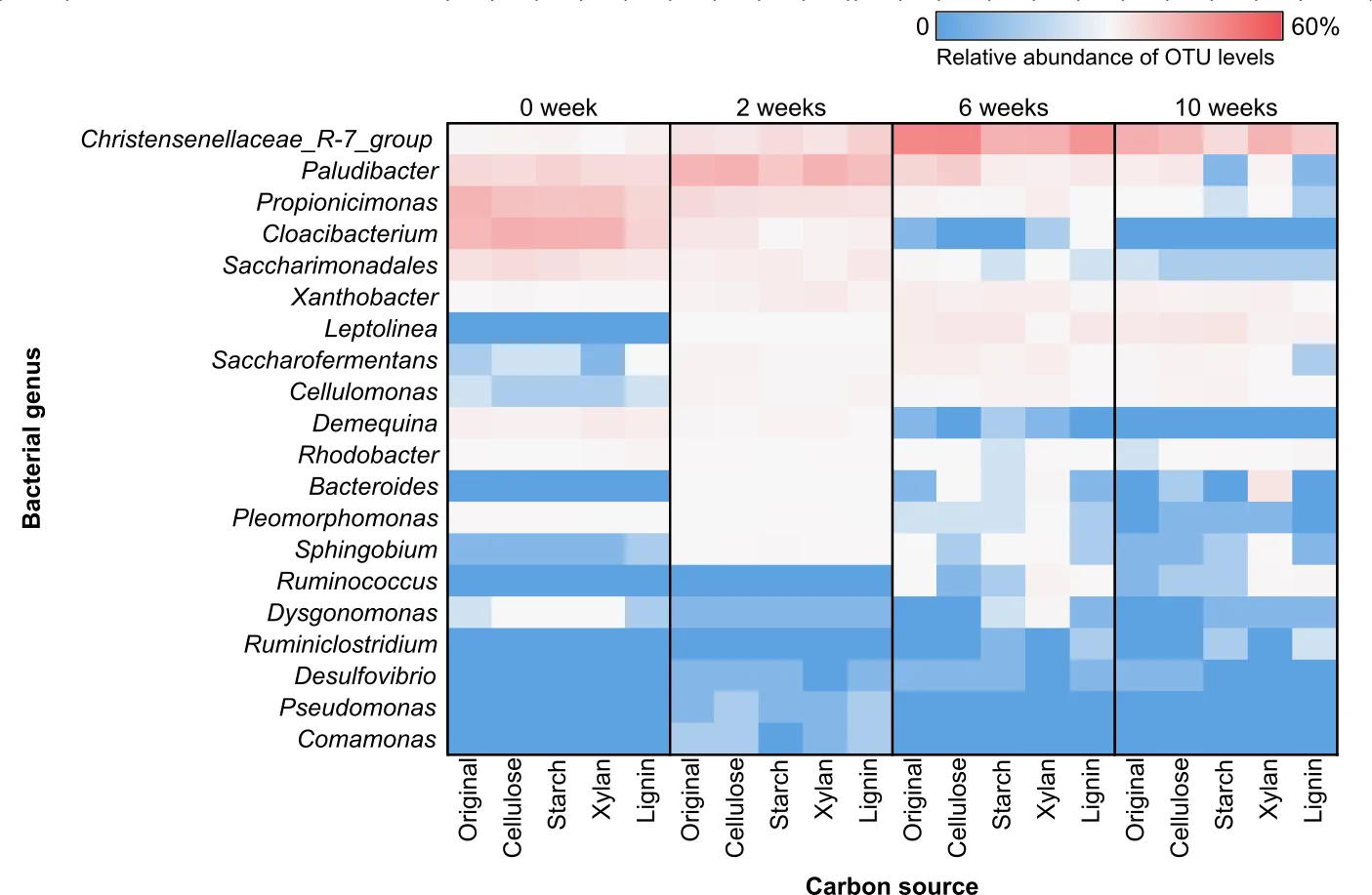
Heatmap illustrating the relative abundance of OTUs of 20 selected dominant bacterial genera in PS, both with and without supplementation with different carbon sources (Original, Cellulose, Starch, Xylan, and Lignin) and at different incubation time points (0, 2, 6, and 10 weeks).

Overall, the changes in bacterial community structure showed the same trend over time for all cultivation conditions, regardless of the carbon source used for supplementation (Fig. 5). These observations were consistent with alpha-diversity results (Fig. 3), which also highlighted the impact of incubation time on bacterial diversity. Even so, the rate of biopolymer removal was affected by some supplementation (Fig. 2), perhaps suggesting that enzymatic activity was enhanced or suppressed even if the bacterial community profile was unaltered. It is possible that the supplemented carbon sources primarily supported a small but active sub-population of the microbiota rather than the dominant genera, which could explain the lack of observable shifts in overall community composition (44). Deeper meta-transcriptomic or meta-proteomic analyses may shed light on these observations in future studies that may also suggest candidate biocatalysts for characterization and deployment in PS hydrolysis studies. Additionally, cross-feeding interactions—where two species feed on metabolites produced by each other (45)—are likely occurring within the PS microbiomes. These interactions are difficult to discern from sequencing data but would contribute to maintaining the stability of the bacterial microbiome.

### Selective enrichment of polymer-degrading bacteria

From an applied perspective, if PS-derived enrichment cultures can easily be generated, these could be used in bioreactors for fermentative sludge minimization or be studied as sources of novel biocatalysts for sludge hydrolysis. Therefore, an enrichment study was performed by sub-culturing the PS microbiome into media providing starch, cellulose, xylan, or lignin as the sole carbon source, followed by incubation for 2 weeks before repeated subculturing into fresh medium with the same carbon source. The M9 minimal medium used supports non-selective cultivation for bacteria, and carbon sources were added to 0.5% (wt/vol). As we were focused on enriching bacteria, only 16S marker gene sequencing was performed. Repeated subculturing in M9 medium without a carbon source resulted in the loss of microbial biomass, and samples from beyond 2 weeks of incubation could not be sequenced due to low DNA yield, confirming that the carbon source carried over from the sludge was depleted quickly, and verifying the selectivity of the other media.

**Fig 6:**
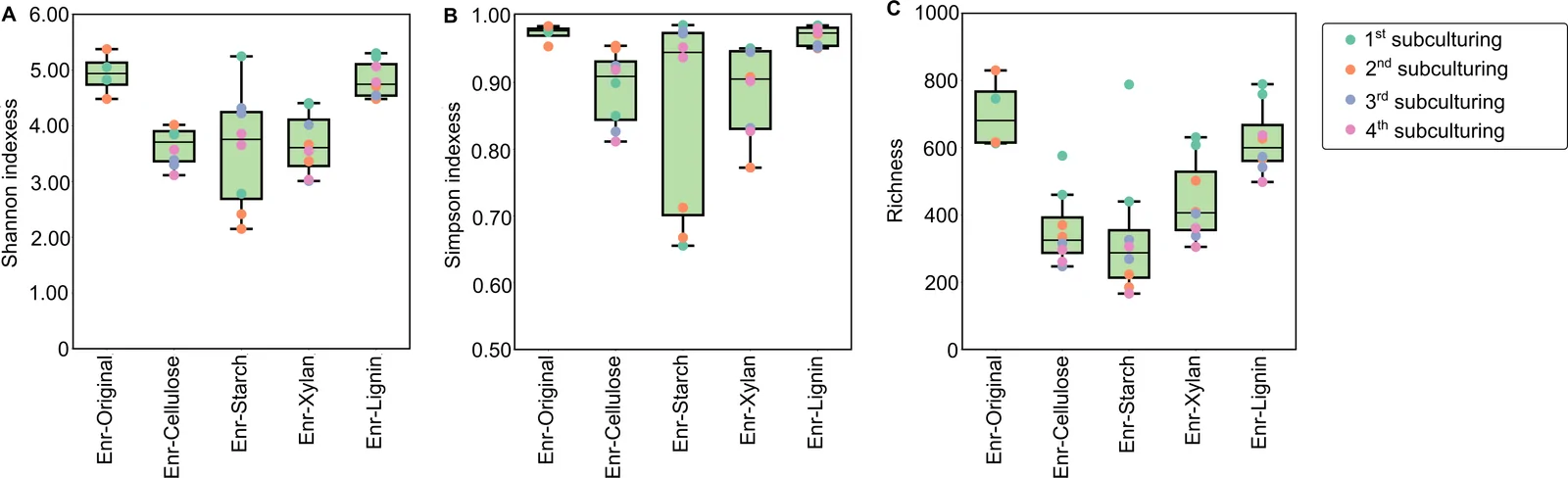
Alpha diversity of bacterial communities (Shannon index, **A**; Simpson index, **B**; Richness, **C**) in enrichment cultures subjected to serial subculturing. The statistical *P* values were shown in supplement data. Note on sample naming: “Enr” stands for enriched cultures; the second part of the sample name indicates the sole carbon source used, except “Enr-Original,” which stands for enriched cultures without any added carbon source.

The Shannon and Simpson indices of enrichment cultures showed high fluctuations throughout the subculturing process (Fig. 6), likely caused by the community needing some time to adapt to a pure carbon source. Despite these fluctuations, the Shannon and Simpson indices still depict an overall trend showing reduced diversity with repeated sub-cultivation steps (Fig. 6A and B). When comparing enriched cultures without any added carbon source, i.e., Enr-Original, the Shannon and Simpson indices show that the provision of cellulose or xylan as sole carbon source significantly decreased bacterial diversity and evenness (Tukey’s HSD *P* value = 0.0107 for cellulose and *P* value = 0.0141 for xylan; Wilcoxon test *P* value = 0.0202 for cellulose and *P* value = 0.0135 for xylan, respectively, Tables S11 and S12). In the case of starch-enriched cultures, the Shannon index showed significantly decreased bacterial diversity (Tukey’s HSD *P* value = 0.0071, Table S11), while the Simpson index presented no significant differences compared to Enr-Original (Wilcoxon test *P* value = 0.3071, Table S12). When lignin was used as the sole carbon source, however, there were no significant differences compared to Enr-Original in either the Shannon or Simpson indices (Tukey’s HSD *P* value = 0.9998, Wilcoxon test *P* value = 0.7587, Tables S11 and S12), likely indicating a failure to specifically enrich for active species.

A clear and significant decreasing trend in richness can be observed across successive sub-cultivations for all substrates (Fig. 6C, ANOVA *P* value = 5.76e-06). This reflects the selective pressure of the substrates and repeated sub-culturing, favoring the growth of specific microbes adapted to these conditions. At the first subculturing, the bacterial richness tended to have greater variability between samples as indicated by wider interquartile ranges, compared to the fourth subculturing, where the community appeared more significantly stabilized (Tukey’s HSD *P* value = 0.00001, Table S13). Moreover, substrate types clearly influenced microbial richness, with cellulose, starch, and xylan generally supporting an elevated abundance of bacterial species, while leading to lower richness compared to the lignin cultivations (Tukey’s HSD *P* value < 0.01, Table S14). This may suggest that a higher number of bacterial species were required to break down the complex structure of lignin, maintaining higher microbial richness in lignin-enriched cultures. More likely, as our data only show proportional abundance, it may indicate that no bacteria were able to grow particularly well on lignin, so there was no outgrowth of successful species. Indeed, fungi are considered the main drivers of lignin degradation in nature, although detailed structural analysis of lignin in our microcosms was not possible in the scope of this study, our crude quantitative analyses indicate that the amount of lignin did not change during complex microcosm incubation (Fig. 2).

**Fig 7:**
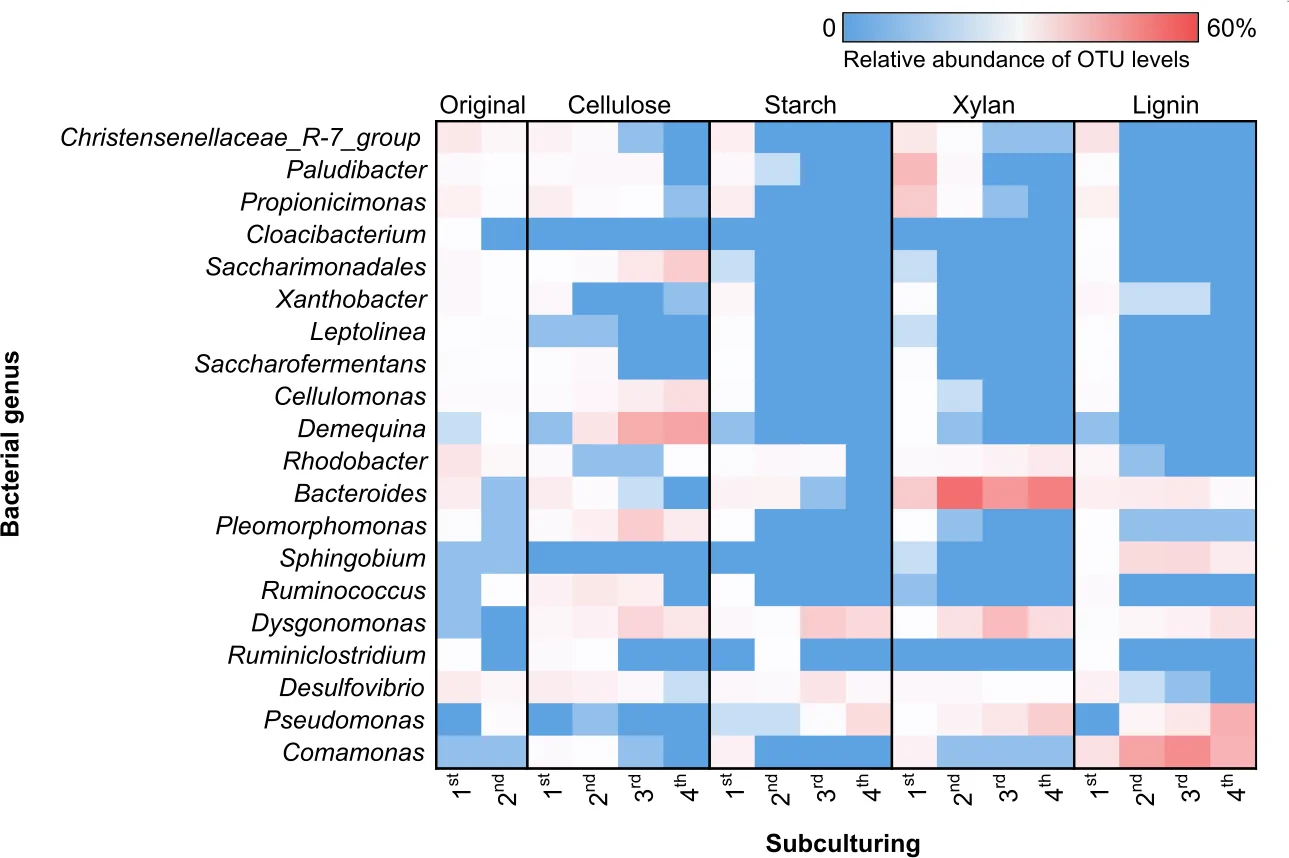
Heatmap illustrates the relative abundance of OTUs of 20 selected dominant genera of bacteria of microbiomes in enriched cultures with serial subculturing.

At the first subculturing, no single genus dominated the enrichment cultures, which still represented an unselected microbial community (Fig. 7). After the second subculturing with starch, xylan, or lignin, the relative abundance of OTUs of many genera dramatically decreased, leaving only a few remaining (Fig. 7). These changes indicated that there was quickly a strong selection for a simplified substrate-adapted microbial sub-community. Interestingly, the cellulose-enriched cultures maintained greater diversity compared to the other enrichments: a heatmap display of the 20 abundant genera of interest in these cultures shows that greater diversity exists after repeated culturing on cellulose compared to the other carbon sources (Fig. 7), perhaps suggesting a larger number of species in the original microbiome are focused on degrading cellulose compared to other biopolymers.

The genera *Saccharimonadales*, *Cellulomonas,* and *Demequina* showed a low relative OTU abundance in complex PS microcosms (Fig. 5) but were highly enriched by pure cellulose provision in M9 medium cultures (Fig. 7). Genera that became abundant in cellulose enrichments might not have been able to compete well in the complex microbiome, even with the addition of 0.5% of cellulose in PS, whereas the cellulose-supplemented M9 medium provided a more optimized nutrient source for specialist cellulolytic bacteria like *Cellulomonas* (46, 47) and *Demequina* (38), as well as the more typical sugar-fermenting genus *Saccharimonadales* (37). As a result, these three genera could proliferate, and their enrichment can be seen in M9 medium containing cellulose as the sole carbon source.

The genus *Bacteroides* is involved in a wide range of functions related to lignocellulose degradation in many diverse ecosystems (48, 49). Although it was not one of the most abundant genera in the PS microcosms, it did become more abundant after 10 weeks of microcosm incubation with xylan supplementation (Fig. 5), suggesting it is capable of xylan degradation in the complex environment. Correspondingly, the genus *Bacteroides* was strongly represented in xylan-fed enrichment cultures (Fig. 7). Within the *Bacteroides* genus, *Bacteroides graminisolvens* was particularly dominant (Table S16); this species has been previously isolated from cattle farming-derived sludge and identified as a xylanolytic bacterium (50), corresponding with its dominance in our xylan-enriched experiments.

The genus *Comamonas* was not particularly abundant in any of the complex microcosm experiments (Fig. 4), but it became dominant in enrichment cultures when exposed to lignin, where the species *Comamonas testosteroni* was particularly significant (Fig. 6; Table S16). Indeed, members of the *Comamonas* genus have previously been used for lignin depolymerization (51–53), and the conversion of lignin-related compounds has been studied in *C. testosteroni* KF-1 (54). To establish a more conclusive connection between the *Comamonas testosteroni* found in this study and lignin degradation, future dedicated work should focus on species isolation and in-depth chemical analysis of lignin degradation.

Interestingly, the genus *Pseudomonas* was abundant in enrichment cultures, including starch, xylan, and lignin, as sole carbon sources, particularly the species *Pseudomonas putida* (Table S16). *Pseudomonas* is a group of Gram-negative bacteria known for its ability to break down a wide variety of organic compounds, including environmental pollutants, and some species play an important role in breaking down lignocellulose (53, 55, 56). In previous studies, the strains *P. putida* (A514) (57), *P. putida* KT2440 (58), and *P. putida* IDPC/pTS110 (59) have been employed for valorizing lignin, while a strain of *Pseudomonas abieticivorans* able to grow on resin acids has been isolated from tree bark sourced from the same pulp mill as was sampled for the current study (19).

### Conclusions

In a first-principles investigation into artificial microcosms based on a pulp and paper sludge waste stream, we have studied biopolymer degradation and changes to microbiomes over time. Our data highlight the interplay between substrate type, incubation time, and microbial adaptation. While major changes in microbial communities due to the supplementation of carbon sources were not observed, shifts in species profile and diversity were observed over time as the composition and structure of the sludge changed. A significant reduction in the biopolymer content of the sludge was observed during incubation in controlled conditions, and this could in part be correlated with changes to the microbiome, as specific genera of bacteria and fungi known for degrading starch, cellulose, xylan, or lignin were identified. As a previously well-validated hydrolytic enzyme cocktail showed poor performance on the sludge waste, our data suggest that native sludge microbes may possess enzyme systems that are better adapted to this complex contaminated substrate, although further investigation is needed. The establishment of enrichment cultures created relevant sub-populations of biomass-degrading bacteria from native PS microbiomes, and these may be of use in the future development of microbial or enzymatic tools for sludge degradation.

## Data Availability

Figures S1 to S3 and Tables S1 to S14 are included in the provided Word document. Tables S15 and S16 (20 selected bacterial genera from microcosm and enrichment experiments) are provided as separate Excel documents. The raw data from all experiments can be found in the separate Excel documents Table S17 (sludge composition and enzymatic hydrolysis analyses) and Table S18 (microbiome sequencing study).

## SUPPLEMENTAL MATERIAL

Supplemental figures and tables spectrum.01359-26-s0001.pdf Figures S1 to S3 and Tables S1 to S14.

Table S15 spectrum.01359-26-s0002.xlsx 20 selected bacterial genera in precipitation sludge microcosms.

Table S16 spectrum.01359-26-s0003.xlsx 20 selected bacterial genera in enrichment cultures.

Table S17 spectrum.01359-26-s0004.xlsx Experimental data used to generate numbers, tables, and figures in the main manuscript that relate to sludge composition and the enzymatic hydrolysis tests.

Table S18 spectrum.01359-26-s0005.xlsx All raw data generated by DNASense that was used to produce the figures and tables in the main manuscript related to microbiome analysis.

ASM does not own the copyrights to Supplemental Material that may be linked to, or accessed through, an article. The authors have granted ASM a non-exclusive, world-wide license to publish the Supplemental Material files. Please contact the corresponding author directly for reuse.
