# Supplementary material for: Exploring the native pulp and paper sludge microbiome to inspire new biotechnologies for waste minimization
Source: Microbiol Spectr. 2026 Jun 9;14(7):e01359-26. doi: 10.1128/spectrum.01359-26 (PMC13339970; doi:10.1128/spectrum.01359-26)
Supplement: Supplemental figures and tables — Figures S1 to S3 and Tables S1 to S14. [file spectrum.01359-26-s0001.pdf]

Supplementary information for:

Exploring the native pulp and paper mill sludge microbiome to inspire new biotechnologies for waste minimisation

Pakinee Thianheng, Kurt Schroeter, Johan Larsbrink, Lauren Sara McKee

A B

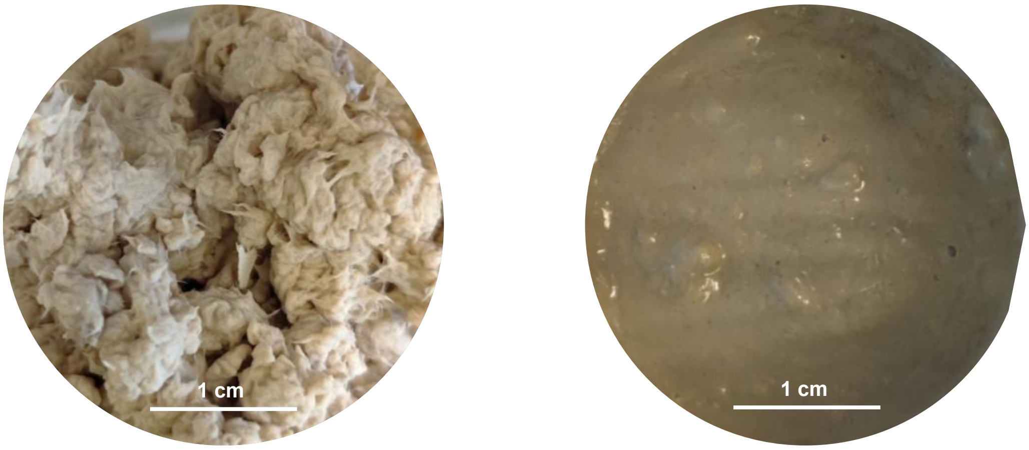

FIG S1 (A) Original precipitation sludge (PS) obtained from a northern Swedish pulp mill was a light brown and heterogeneous, fibrous material with a moisture content of 66%, giving it a semi-solid consistency. (B) After 10 weeks, PS supplemented with lignin was observed to have some visible bubbles, a bad odour, and a more dissolved appearance, indicating that absorptive polymeric fibres have been converted to smaller fragments of higher solubility. PS supplemented with other carbon sources also became more homogenous and dissolved, though to a lesser extent.

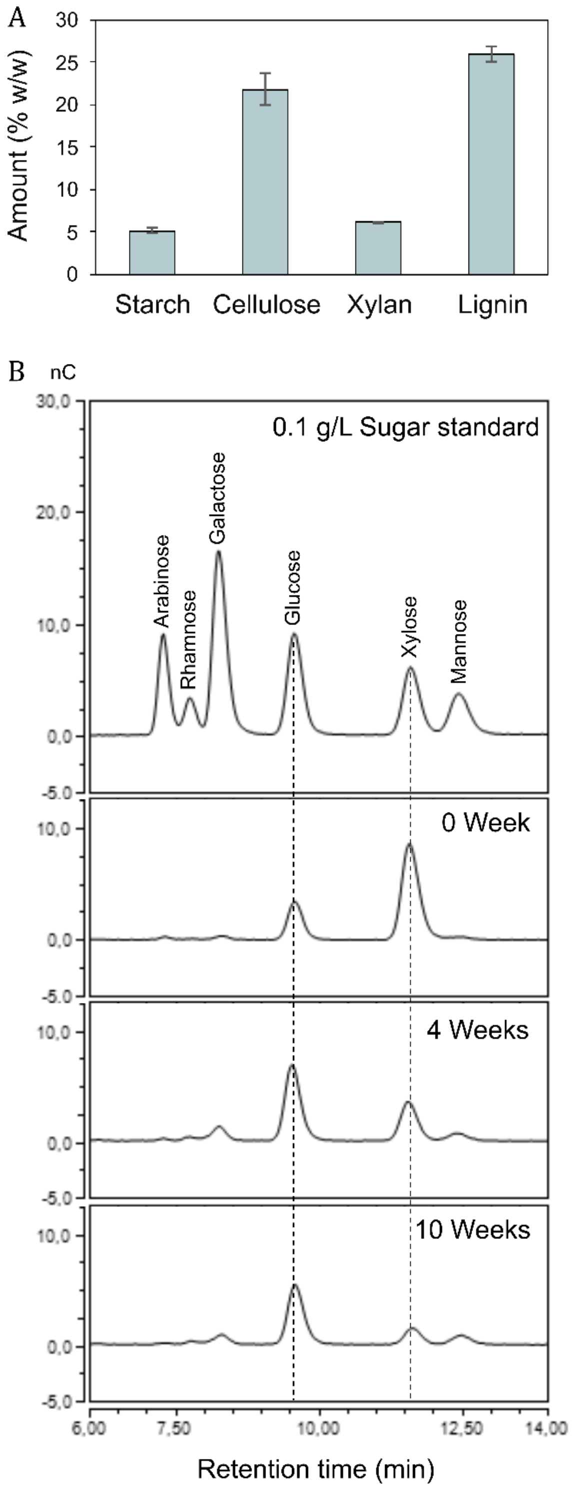

FIG S2 (A) Organic compositions in original PS. The bar graph represents the mean of four replicates, with error bars indicating standard deviations (SD). (B) Representative example of HPAEC-PAD spectra of sugar analysis of PS-Original after incubating for 0, 4, and 10 weeks, compared with 0.1 g/L standards of arabinose, rhamnose, galactose, glucose, xylose, and mannose.

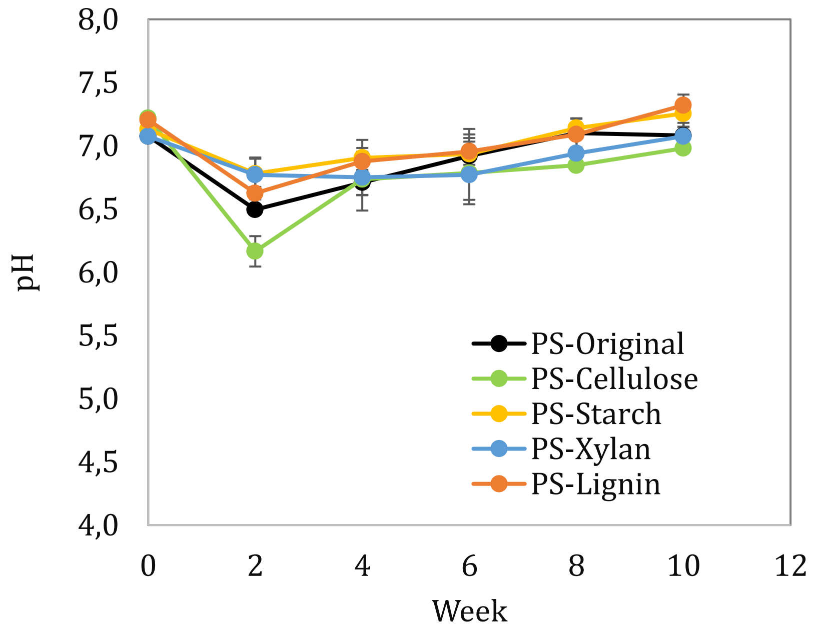

FIG S3 pH changes in PS microcosms supplemented with different carbon sources during a 10-week incubation. Data are shown as averages from samples that were analysed in triplicate and error bars indicate standard deviation.

Table S1 Statistical analysis of the Shannon index of bacteria communities compared between carbon sources at week 0. A Kruskal-Wallis test presented no significant difference between carbon sources at 0 week with *p*-value = 0.448. The *p*-values of pairwise comparisons presented in the table were compared using the Wilcoxon test of different carbon source-supplemented conditions at 0 week. The *p*-values greater than 0.05 indicate no statistically significant difference.

	PS-Cellulose	PS-Lignin	PS-Original	PS-Starch
PS-Lignin	0.64	-	-	-
PS-Original	0.80	0.64	-	-
PS-Starch	0.64	0.72	0.64	-
PS-Xylan	0.88	0.64	0.80	0.64

Table S2 Statistical analysis of the Shannon index of bacteria communities compared between carbon sources at week 10. A Kruskal-Wallis test presented no significant difference between carbon sources at week 10 with *p*-value = 0.359. The *p*-values of pairwise comparisons presented in the table were compared using the Wilcoxon test of different carbon source-supplemented conditions at 10 weeks. The *p*-values greater than 0.05 indicate no statistically significant difference.

	PS-Cellulose	PS-Lignin	PS-Original	PS-Starch
PS-Lignin	0.83	-	-	-
PS-Original	0.95	0.83	-	-
PS-Starch	0.83	0.95	0.83	-
PS-Xylan	1.00	1.00	1.00	0.95

Table S3 Statistical analysis of the Shannon index of bacteria compared between incubation times. A Kruskal-Wallis test presented a significant difference between incubation time with *p*-value = 0.0002. The *p*-values of pairwise comparisons presented in the table were compared using the Wilcoxon test of different incubation times. The *p*-values greater than 0.05 indicate no statistically significant difference.

	At 0 weeks	At 10 weeks	At 2 weeks
At 10 weeks	0.0010	-	-
At 2 weeks	0.0010	0.0042	-
At 6 weeks	0.0058	0.1262	0.7394

Table S4 Statistical analysis of the Simpson index of bacteria communities compared between incubation time. A Kruskal-Wallis test presented a significant difference between incubation time with *p*-value = 2.964e-05, but no significant difference between carbon sources with *p*-value = 0.5977. The *p*-values of pairwise comparisons presented in the table were compared using the Wilcoxon test of different incubation times. The *p*-values greater than 0.05 indicate no statistically significant difference.

	At 0 weeks	At 10 weeks	At 2 weeks
At 10 weeks	0.0006	-	-
At 2 weeks	0.0010	0.0082	-
At 6 weeks	0.0006	0.0031	0.6842

Table S5 Statistical analysis of the Chao 1 index of bacteria communities compared between incubation times. An ANOVA test presented a significant difference between incubation times (ANOVA, *p*-value = 0.0413), but no significant differences between carbon sources (ANOVA, *p*-value = 0.3487). The *p*-values of pairwise comparisons presented in the table were compared using the Tukey's HSD test of different incubation times. The *p*-values greater than 0.05 indicate no statistically significant difference.

	At 0 weeks	At 10 weeks	At 2 weeks
At 10 weeks	0.0248	-	-
At 2 weeks	0.4112	0.4268	-
At 6 weeks	0.2919	0.5677	0.9948

Table S6 Statistical analysis of the Shannon index of fungal communities compared between carbon sources at week 0. An ANOVA test presented no significant differences between carbon sources (ANOVA, *p*-value = 0.774). The *p*-values of pairwise comparisons presented in the table were compared using the Tukey's HSD test of different carbon source-supplemented conditions at 0 week. The *p*-values greater than 0.05 indicate no statistically significant difference.

	PS-Cellulose	PS-Lignin	PS-Original	PS-Starch
PS-Lignin	1.00	-	-	-
PS-Original	1.00	1.00	-	-
PS-Starch	1.00	1.00	1.00	-
PS-Xylan	1.00	1.00	1.00	1.00

Table S7 Statistical analysis of the Shannon index of fungal communities compared between carbon sources at week 10. An ANOVA test presented no significant differences between carbon sources (ANOVA, *p*-value = 0.774). The *p*-values of pairwise comparisons presented in the table were compared using the Tukey's HSD test of different carbon source-supplemented conditions at week 10. The *p*-values greater than 0.05 indicate no statistically significant difference.

	PS-Cellulose	PS-Lignin	PS-Original	PS-Starch
PS-Lignin	1.00	-	-	-
PS-Original	1.00	1.00	-	-
PS-Starch	1.00	1.00	1.00	-
PS-Xylan	1.00	1.00	1.00	1.00

Table S8 Statistical analysis of the Shannon index of fungal communities compared between incubation times. An ANOVA test presented a significant difference between incubation times (ANOVA, *p*-value = 5.8e-09). The *p*-values of pairwise comparisons presented in the table were compared using the Tukey's HSD test of different incubation times. The *p*-values greater than 0.05 indicate no statistically significant difference.

	At 0 weeks	At 10 weeks	At 2 weeks
At 10 weeks	0.0000	-	-
At 2 weeks	0.0002	0.0001	-
At 6 weeks	3.0e-07	0.0889	0.0359

Table S9 Statistical analysis of the Simpson index of fungal communities compared between incubation times. A Kruskal-Wallis test presented a significant difference between incubation times (Kruskal-Wallis, *p*-value = 1.791e-05), but no significant differences between carbon sources (Kruskal-Wallis, *p*-value = 0.9979). The *p*-values of pairwise comparisons presented in the table were compared using the Wilcoxon test of different incubation times. The *p*-values greater than 0.05 indicate no statistically significant difference.

	At 0 weeks	At 10 weeks	At 2 weeks
At 10 weeks	0.0003	-	-
At 2 weeks	0.0103	0.0010	-
At 6 weeks	0.0010	0.0630	0.0176

Table S10 Statistical analysis of the Chao 1 index of fungal communities compared between incubation times. An ANOVA test presented a significant difference between incubation times (ANOVA, *p*-value = 1.39e-13), but no significant differences between carbon sources (ANOVA, *p*-value = 0.0702). The *p*-values of pairwise comparisons presented in the table were compared using the Tukey's HSD test of different incubation times. The *p*-values greater than 0.05 indicate no statistically significant difference.

	At 0 weeks	At 10 weeks	At 2 weeks
At 10 weeks	0.0000	-	-
At 2 weeks	0.0000	0.0004	-
At 6 weeks	0.0000	0.3756	0.0152

Table S11 Statistical analysis of the Shannon index of enriched cultures compared between carbon sources. An ANOVA test presented a significant difference between carbon sources (ANOVA, *p*-value = 7.04e-05). The *p*-values of pairwise comparisons presented in the table were compared using the Tukey's HSD test of different carbon sources. The *p*-values greater than 0.05 indicate no statistically significant difference.

	Enr-Cellulose	Enr-Lignin	Enr-Original	Enr-Starch
Enr_Lignin	0.0011	-	-	-
Enr_Original	0.0107	0.9998	-	-
Enr_Starch	0.9993	0.0007	0.0071	-
Enr_Xylan	0.9998	0.0016	0.0141	0.9942

Table S12 Statistical analysis of the Simpson index of enriched cultures compared between carbon sources. A Kruskal-Wallis test presented a significant difference between carbon sources (Kruskal-Wallis, *p*-value = 0.002822). The *p*-values of pairwise comparisons presented in the table were compared using the Wilcoxon test of different carbon sources. The *p*-values greater than 0.05 indicate no statistically significant difference.

	Enr-Cellulose	Enr-Lignin	Enr-Original	Enr-Starch
Enr_Lignin	0.0031	-	-	-
Enr_Original	0.0202	0.7587	-	-
Enr_Starch	0.7587	0.3248	0.3071	-
Enr_Xylan	0.9591	0.0016	0.0135	0.7587

Table S13 Statistical analysis of the Chao1 index of enriched cultures compared between subculturing. An ANOVA test presented a significant difference between subculturing (ANOVA, *p*-value = 40e-06). The *p*-values of pairwise comparisons presented in the table were compared using the Tukey's HSD test of subculturing. The *p*-values greater than 0.05 indicate no statistically significant difference.

	1st subculturing	2nd subculturing	3rd subculturing
2nd subculturing	0.0014	-	-
3rd subculturing	0.00002	0.1669	-
4th subculturing	0.00001	0.0595	0.9506

Table S14 Statistical analysis of the Chao 1 index of enriched cultures compared between carbon sources. An ANOVA test presented a significant difference between carbon sources (ANOVA, *p*-value = 5.76e-06). The *p*-values of pairwise comparisons presented in the table were compared using the Tukey's HSD test of different carbon sources. The *p*-values greater than 0.05 indicate no statistically significant difference.

	Enr-Cellulose	Enr-Lignin	Enr-Original	Enr-Starch
Enr_Lignin	0.0001	-	-	-
Enr_Original	0.0015	0.9975	-	-
Enr_Starch	0.9906	0.00003	0.0007	-
Enr_Xylan	0.3000	0.0050	0.0470	0.1434
